# Action versus valence in decision making

**DOI:** 10.1016/j.tics.2014.01.003

**Published:** 2014-04

**Authors:** Marc Guitart-Masip, Emrah Duzel, Ray Dolan, Peter Dayan

**Affiliations:** 1 Aging Research Centre, Karolinska Institute, SE-11330 Stockholm, Sweden; 2 Wellcome Trust Centre for Neuroimaging, Institute of Neurology, University College London, London WC1N 3BG, UK; 3 Institute of Cognitive Neuroscience, University College London, London WC1N 3AR, UK; 4 Otto von Guericke University Magdeburg, Institute of Cognitive Neurology and Dementia Research, D-39120 Magdeburg, Germany; 5 German Center for Neurodegenerative Diseases, D-39120 Magdeburg, Germany; 6 Gatsby Computational Neuroscience Unit, University College London, London W1CN 3AR, UK

**Keywords:** Pavlovian, instrumental, action, value, striatum, dopamine

## Abstract

The selection of actions, and the vigor with which they are executed, are influenced by the affective valence of predicted outcomes. This interaction between action and valence significantly influences appropriate and inappropriate choices and is implicated in the expression of psychiatric and neurological abnormalities, including impulsivity and addiction. We review a series of recent human behavioral, neuroimaging, and pharmacological studies whose key design feature is an orthogonal manipulation of action and valence. These studies find that the interaction between the two is subject to the critical influence of dopamine. They also challenge existing views that neural representations in the striatum focus on valence, showing instead a dominance of the anticipation of action.

## Highlights

• Pavlovian responses couple action and valence.
• This coupling interferes with instrumental learning and performance.
• Action dominates valence in the striatum and dopaminergic midbrain.
• Boosting dopamine enhances the dominance of action over valence in the striatum.
• Boosting dopamine decreases the extent of the behavioral coupling between action and valence.

## Introduction

**Figure 1:**
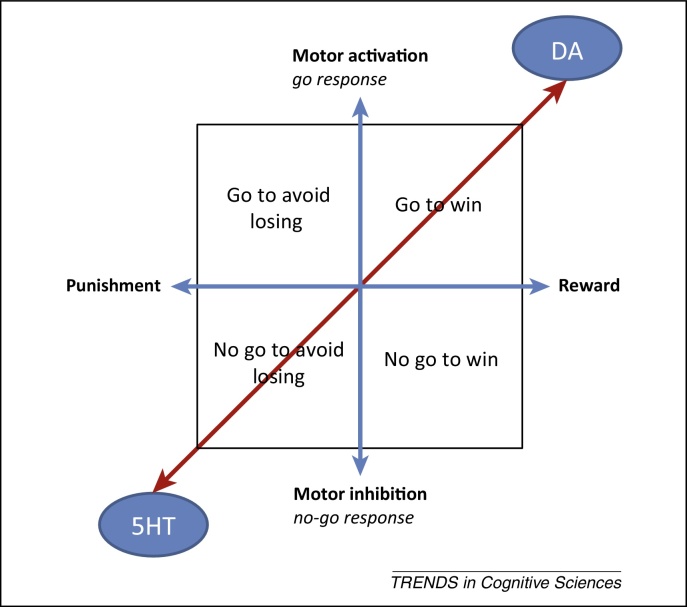
The two axes of behavioral control – affect or valence – running from punishment to reward, and effect or action, running from motor inhibition to motor activation. For an instrumental control system (in blue) these two axes are mutually independent. Therefore, an instrumental controller should learn equally well to invigorate action to obtain a reward (‘go to win’), to invigorate action to avoid a punishment (‘go to avoid losing’), to inhibit action to avoid punishment (‘no go to avoid losing’), and to inhibit action to obtain a reward (‘no go to win’). By contrast, action and valence are coupled in a Pavlovian control system (in red) so that reward is associated with action invigoration (approach and engagement) whereas punishment is associated with action inhibition (withdrawal and inhibition). At the neuronal level, the same dual association between action and valence may be observed within ascending monoaminergic systems. The dopaminergic system (DA) is involved in generating active motivated behavior and reward prediction, whereas the serotonergic system (5HT) appears affiliated with behavioral inhibition, potentially in aversive contexts.

Subjects should rationally choose which actions to emit (see Glossary), and with what vigor, based on the rewards or punishment potentially gained or avoided. Actions are thus instructed by valence. Because subjects might just as well act vigorously or withhold a response studiously to gain a reward or avoid a punishment, there should be no *a priori* dependence between action and valence (Figure 1) and they are duly studied mostly in isolation. This research has revealed that, among other regions, supplementary motor cortex and sensorimotor sectors of the basal ganglia are involved in controlling motor performance 1, 2, 3, 4 whereas the ventral striatum and its dopaminergic innervation, as well as medial and orbital prefrontal regions, are associated with the representation and calculation of expected affective value 5, 6, 7, 8, 9.

This *a priori* independence between action and valence is gracefully satisfied in instrumental control 10, 11. However, instrumental control competes and cooperates with Pavlovian control, which generates prespecified responses in the light of biologically significant outcomes and their predictors. Pavlovian responses couple action and valence (promoting approach and engagement in the face of reward and inhibition or perhaps active escape in the face of punishment), forcing interactions even when they are suboptimal. The importance of these interactions is that they might help explain a wealth of behavioral anomalies such as impulsivity and addiction, where the effect of valence apparently overrides instrumental action selection 12, 13, 14.

It is perhaps surprising that, until recently, only a few tasks have examined these interactions (e.g., 15, 16). For example, most imaging tasks that study the representation of valence in the human brain have either required or at least permitted particular actions. However, in such designs it is not possible to distinguish whether a signal is associated with valence (i.e., reward versus punishment) or action (i.e., go versus no-go). Thus, a fuller psychological and neural understanding depends on the simultaneous and separate manipulation of these factors.

One task (which we call the ‘orthogonalized go/no-go task’) that was explicitly designed to study the interaction of action and valence [17] involves four conditions, each signaled by a different visual stimulus (Figure 1): active responses to obtain rewards (‘go to win’); active responses to avoid punishment (‘go to avoid losing’); passive responses to obtain rewards (‘no go to win’); and passive responses to avoid punishment (‘no go to avoid losing’). Variants have been used to study behavior and brain responses [as measured with functional MRI (fMRI)] when subjects were either explicitly instructed what to do or had to learn this for themselves. The effects of manipulating the dopaminergic and serotonergic systems with systemic manipulations have also been examined 17, 18, 19, 20, 21, 22.

The results of this substantial body of experiments pose two challenges. First, they reveal that the strength of coupling between valence and action has particular consequences for learning. This in turn highlights the importance of orthogonalizing them to elucidate cognitive and neuronal aspects of value representation and action selection. Second, these results indicate limits to a dominant view of the striatum that has emerged from neuroimaging; namely, that it preferentially encodes valence. Instead, these studies strongly support the idea that the striatum encodes a tendency toward action. The experiments also highlight two distinct contributions of dopaminergic neuromodulation: the control of motivation in instrumental responding and the extent to which action and valence interact to influence behavior.

## Behavioral interactions between action and valence

When carefully instructed on the contingencies of the task, and given ample practice, subjects correctly choose go/no-go regardless of valence on more than 95% of trials. Nevertheless, anticipating punishment impairs performance of well-learned instrumental choices by slowing go responses 19, 20, revealing the essential interaction between action and valence akin to conditioned-suppression experiments. Similarly, Crockett and colleagues showed that correct go responses are slower when feedback involves punishment 17, 22.

Further, when participants learn the action contingencies by trial and error, go performance is better when it leads to reward and no-go performance for punishment omission [21]. These results have been replicated in independent samples 18, 23 and in healthy older adults [24]. Critically, some participants perform substantially worse than chance in the no go to win condition in which Pavlovian and instrumental systems conflict. This is a human analog of an omission schedule [12] and suggests the danger of overlooking Pavlovian influences in seemingly straightforward instrumental contexts.

The most parsimonious of a nested sequence of reinforcement learning models that parameterize alternative accounts of this behavior (Box 1) recapitulates the learning asymmetry (relatively impaired learning in ‘go to avoid losing’ and ‘no go to win’ compared with ‘go to win’ and ‘no go to avoid losing’, respectively) by specifying an interaction between instrumental and Pavlovian control mechanisms [21]. In essence, the latter promotes or inhibits go choices in the winning and losing conditions, respectively.

Box 1 **Computational modeling of the learning behavior**

We built several nested models incorporating different instrumental and Pavlovian reinforcement-learning hypotheses. All models were fitted to the observed behavioral data and compared using Bayesian Information Criteria (BIC). All models learned separate propensities $w(a_{t},s_{t})$ for action *a*_*t*_ (go or no-go) on trial *t* under condition *s*_*t*_. The model assigned probabilities to each action using a sigmoid function. The base model (RW) was purely instrumental: *w*(*a*,*s*) = *Q*(*a*,*s*). Action values *Q*(*a*,*s*) were learned independently of the valence of the outcomes using the Rescorla–Wagner rule:

(I) $$Q_{t}(a_{t},s_{t})=Q_{t-1}(a_{t},s_{t})+\varepsilon (\rho r_{t}-Q_{t-1}(a_{t},s_{t}))$$

where *ɛ* is the learning rate. Reinforcements enter the equation through *r*_*t*_∈{-1,0,1} and *ρ* is a free parameter that determines the effective size of reinforcements.

This model was augmented in successive steps. In RW + noise, the model included irreducible choice noise in the instrumental system by squashing the sigmoid function:

(II) $$p(a_{t}|s_{t})=\left[\frac{\exp(w(a_{t},s_{t})}{\sum_{a'}\exp(w(a',s_{t}))}\right](1-\xi )+\frac{\xi }{2}$$

where ξ is the noise parameter, which was free to vary between 0 and 1.

In RW + noise + bias, the model further included a value-independent and static action bias *b* that promotes or suppresses go choices equally in all conditions:

(III) $$w_{t}(a,s)=\left\{\begin{array}{cc} Q_{t}(a,s)+b & if\;a=go\\ Q_{t}(a,s) & else \end{array}.\right)$$

In RW + noise + bias + Pav, the model also included a (Pavlovian) parameter π that adds a fraction of the state value *V*(*s*) into the action values learned by the instrumental system, thus effectively coupling action and valence during learning, promoting a go choice when *V*(*s*) is positive and no-go choice when *V*(*s*) is negative:

(IV) $$w_{t}(a,s)=\left\{\begin{array}{ll} Q_{t}(a,s)+b+\pi V_{t}(s) & if\;a=go\\ Q_{t}(a,s) & else \end{array}.\right)$$

(V) $$V_{t}(s_{t})=V_{t-1}(s_{t})+\varepsilon (\rho r_{t}-V_{t-1}(s_{t}))$$

We also considered the possibility that behavioral asymmetries arise because of differences in reward and punishment sensitivities. Thus, in RW(rew/pun) + noise + bias, the instrumental system included separate reward and punishment sensitivities allowing different values of the parameter *ρ* on reward and punishment trials.

*Observed and modeled behavior in the orthogonalized go/no-go task* (Figure I)

**Figure I:**
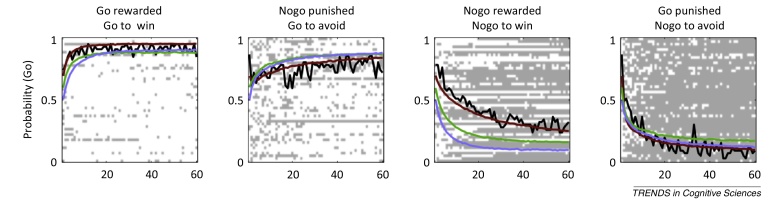
shows the learning time courses for all four conditions of our task. Each row of the raster images shows the choices of one of the 47 subjects in each of the four conditions. Go responses are depicted in white and no-go responses are depicted in grey. The overlaid black lines depict the time-varying probability, across subjects, of making a go response. The colored lines show the same time-varying probability, but evaluated on choices sampled from the model (blue for RW + noise; green for RW + noise + bias; brown, the winning model, for RW + noise + Pav). Adapted from [21].

There is a formal similarity between these results and those of Pavlovian to Instrumental transfer (PIT) tasks in animals and humans, which have previously demonstrated an interaction between Pavlovian and instrumental control 25, 26, 27, 28, 29, 30. As here, go responses that result in an instrumental approach are promoted or inhibited by appetitive- or aversive-predicting stimuli, respectively. However, a notable difference is that, in PIT paradigms, the two sorts of conditioning are taught separately. The orthogonalized go/no-go task demonstrates a severe disruption of learning due to the direct coupling of action and valence characteristic of Pavlovian responses in a simpler task that lacks any such partition.

Why might such striking interactions between action and valence exist despite being deleterious? Pavlovian actions have plausibly been perfected in ancestral environments as hard-wired knowledge of good behavioral responses; for instance, given mortal threats [12]. That such a prior can sometimes delay learning and inhibit performance in unusual environments should not detract from its huge advantage in obviating learning.

What might underlie these behavioral results? Existing neuroimaging studies that include only conditions where reward is anticipated along with action are difficult to interpret unambiguously. Thus, an important next step is to scan human volunteers using fMRI while they perform the orthogonalized go/no-go task.

## Neural representations of action and valence

fMRI allows assessment of the joint contribution to neural responses of the anticipation of action or inaction and the valence of potential outcomes independently of motor performance or outcome delivery. Ample data suggest that blood oxygenation level-dependent (BOLD) signals in the striatum at choice correlate with the action values of the chosen options 31, 32; thus one might expect a valence-dominated signal during anticipation. Furthermore, the Pavlovian influences implied by the best-fitting model (Box 1) might naturally be expected to modulate these prediction and prediction error signals. Surprisingly, neither effect emerges; in fact, no significant neural correlate of the strong interaction evident in behavior is apparent, except that only those participants who learn all task conditions well, and thus overcome the Pavlovian bias, show increased BOLD responses in the inferior frontal gyrus in trials requiring motor inhibition [21].

**Figure 2:**
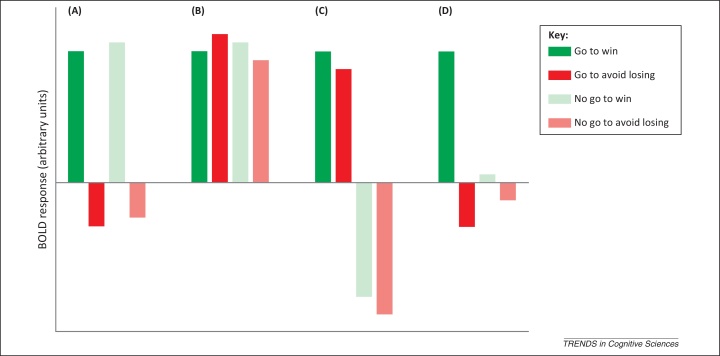
Expected and observed blood oxygenation level-dependent (BOLD) responses in the striatum and the SN/VTA. This figure shows abstract representations of the key signals in anticipatory BOLD responses that is expected within the striatum and substantia nigra pars compacta/ventral tegmental area (SN/VTA) according to prevailing theories **(A–B)** and compared with the signals actually observed when action and valence were manipulated within the same experiment **(C–D)**. Filled colors represent the go conditions and transparent colors the no-go conditions. The winning conditions are represented in green and the avoid-losing conditions in red. (A) Predicted observations: Reward-prediction error. If a brain region reports a reward-prediction error, one should observe a main effect of valence because cues predicting wins would lead to positive values and cues predicting losses would lead to negative values, regardless of action requirements. (B) Predicted observations: Salience. If a brain region reflects salience, one should observe a BOLD response of equivalent magnitude in all conditions of our task, because punishments, rewards, and prediction errors associated with both of these should be reported with the same sign. (C) Actual observations: Main effect of action. BOLD responses during anticipation were higher when an action needed to be performed than when an action needed to be withheld. Importantly, the BOLD response to the ‘go to win’ condition was higher than the BOLD response to the ‘no go to win’ condition despite both conditions being associated with the same expected value. This was the most pervasive signal in the experiments, evident across the striatum (dorsal and ventral) and the SN/VTA. (D) Actual observations: Action-dependent reward-prediction error. A main effect of valence was observed only when an action was required in a location within the ventral striatum compatible with the nucleus accumbens. This signal was restricted to the ventral striatum and survived correction for multiple comparisons only in an experiment with a large sample size (*N* = 54).

Instead, BOLD responses in the striatum and substantia nigra pars compacta/ventral tegmental area (SN/VTA) are dominated by action requirements (Figure 2A) 19, 20. Importantly, despite ‘go to win’ and ‘no go to win’ conditions signaling the same expected value (and thus salience), striatal BOLD responses are reliably higher in the ‘go to win’ condition. Similar results have been described in the ventral striatum when comparing active and passive avoidance of punishment [33]. Interestingly, electrophysiological evidence in rats also shows contrasting neuronal responses in the nucleus accumbens to cues instructing a go or a no-go response despite both cues signaling the equivalent reward [34]. However, in the latter case, there was more activity for ‘no go to win’ than for ‘go to win’. Further, in the learning version of the task, BOLD responses in the striatum and the SN/VTA track instrumental action values with a positive and negative relationship between value and brain activity for go and no-go, respectively [21]. Subsidiary modulation of BOLD responses according to valence does not survive multiple comparisons even when restricted to just the ventral striatum [19]. Even in an expanded dataset, the influence of valence remains significant in go conditions only, with higher BOLD responses in the ‘go to win’ condition than in ‘go to avoid losing’ [20].

The task design precludes a detailed study of BOLD responses to outcomes. However, as expected (see [5] for a review), responses in the ventral striatum and medial prefrontal cortex are significantly greater for winning compared with losing. Winning after go is not different from winning after no-go. However, this has been observed when go responses are more physically demanding, suggesting that the costs associated with the performance of an action may have a role in the processing of outcomes 35, 36.

In brief, these data demonstrate that, during the anticipatory phase and before any action is performed or outcome realized, the coding of action requirements dominates the coding of valence or expected value in the striatum and SN/VTA. What neural systems might contribute to this? Obvious candidates include ascending monoaminergic dopamine and serotonin systems [37]. The dopamine system is involved in generating active motivated behavior 38, 39 and instrumental learning through reward-prediction errors [40]. The serotonin system appears affiliated with behavioral inhibition in aversive contexts 17, 22, 41. A logical step is to manipulate these systems pharmacologically while subjects perform the orthogonalized go/no-go task.

## Pharmacological modulations of the interactions between action and valence

In the instructed version of the task, boosting central dopamine levels in young, healthy participants, via systemic administration of levodopa leads to faster motor responses in both go conditions. However, neuronal effects depend on valence, with increased activity for anticipated go versus no-go choices in the striatum and SN/VTA only when the potential outcome is a reward [20]. Only in the striatum does boosting dopamine decrease the overall striatal BOLD signal for the ‘no go to win’ condition. Remarkably, in a learning experiment, boosting dopamine levels decreased the coupling between action and valence compared with placebo [18], with smaller differences in performance between the two go conditions or between the two no-go conditions. In other experiments studying the role of serotonin, the only significant effect of decreasing central serotonin levels with acute tryptophan depletion is to abolish the effects of anticipatory punishment on the vigor of the go responses 17, 22.

The whole collection of results colors our picture of the striatum and the impact of dopamine away from pure valence – as, for instance, in reward-prediction errors that train predictions – and toward action invigoration. We next examine these two characteristics, along with the final effect of dopamine on reducing the coupling between the two.

## Reward-prediction errors

The dominance of action over valence in striatal and SN/VTA BOLD responses during anticipation (and before execution), as observed in our imaging experiments, challenges conventional assumptions regarding the basal ganglia. This finding may appear obvious given the predominance of motor symptoms in basal ganglia disorders such as Huntington's and Parkinson's disease [42]. However, electrophysiological and voltammetry examinations of the responses of dopamine neurons 40, 43, 44 and striatal dopamine transients 45, 46 and a wealth of fMRI experiments on state-based 47, 48 and action-based 49, 50 values, as well as effort-based cost–benefit evaluation [51], all point to a main effect of valence, via the temporal-difference reward-prediction error, and not a main effect of action (Figure 2).

Note, however, that most experiments reporting reward-prediction errors require a choice between actions (rather than between action and inaction). Even popular Pavlovian paradigms compare rewarded actions with unrewarded foil actions or require (or engender) menial movements (e.g., 47, 48, 52). Interestingly, the weak influence of valence we observed was entirely conditional on a requirement for action and was not modulated by levodopa [20]. However, as a null result this must be interpreted with caution and does not provide evidence that the representation of valence in the striatum is independent of dopamine.

Dopamine has been implicated in two relevant processes beyond its phasic signaling of a reward-prediction error 43, 53. First, it has a prime role in the generation and invigoration of motor responses, including instrumental and Pavlovian actions directed to rewards and punishments 38, 39. This involvement in action invigoration, regardless of valence, resonates with the dominance of action over valence observed at the neuronal level. Second, dopamine also supports high-level cognitive functions such as working memory [54] and long-term memory [55], functions that may be critical for learning the contingencies of the orthogonalized go/no-go task.

## Dopamine and action invigoration

A role for dopamine in appetitive invigoration is apparent in the decreased motor activity or motivation to work for rewards 39, 56 following dopamine depletion or the increased vigor in appetitive PIT [25] when dopamine is enhanced in the nucleus accumbens. It is associated with the dopamine incentive salience hypothesis [57], which has been observed to be in consilience with temporal-difference prediction-error coding [58]. In our task, when dopamine is enhanced systemically, bolstered brain representations of rewarded action and invigorated instrumental responding regardless of valence are observed [20], the latter possibly mediated by the effects of an assumed elevated average reward rate [59].

However, perhaps the best test bed for this role of dopamine is active avoidance, as when performing a go action to avoid losing. According to two-factor theories of active avoidance 60, 61, actions that change the state of the environment and so abolish the possibility of a loss are reinforced by a consequential attainment of safety. These actions are duly associated with a positive prediction error (going from a negative value to zero). If this prediction error is represented by dopaminergic activity ‘go to avoid losing’ actions would ultimately have similar instrumental status to ‘go to win’ actions (consistent with their similar representation in striatal BOLD responses).

Dopamine's involvement in aversion and action inhibition is complex. There is evidence that dopamine is released in punishing conditions [39] and is involved in performing active avoidance responses 62, 63. Phasic responses in dopamine neurons to aversive stimuli are widely reported 64, 65, 66, 67, 68. Moreover, fMRI experiments have shown evoked striatal BOLD signals of equal magnitude to reward and punishment anticipation 15, 16, 69 when an action is required 19, 20, 33, as well as a correlation with aversive prediction errors in the striatum 6, 70, 71, 72. Furthermore, dopamine depletion impairs acquisition of active avoidance [62], as has long been described in the animal pharmacology literature [39]. In the two-factor account, avoidance of a potential punishment is coded as a reward and thus may contribute to the average reward rate and invigoration of instrumental action. Note, however, that the relation between average reward rate and invigoration of action is not identical in rewarding and punishing contexts because the two contexts differ in terms of the Pavlovian responses [73].

Altogether the findings fit with a suggested role for dopamine in modulating vigor or motivation for actions independent of valence. The striatum would signal the propensity to perform an action largely independent of state values, as in original accounts of the actor in an actor–critic architecture [74]. Note that salience accounts of dopamine gains no succor from these findings because, to the extent that striatal BOLD signal is seen as an indirect report of dopamine release, they are inconsistent with the finding that responses to reward- and punishment-predictive cues are markedly different dependent on whether an active response is required (Figure 2).

Of course, two-factor theories pose an extra requirement. ‘Go to avoid losing’ actions differ from ‘go to win’ actions in that the negative valence of the unsafe state must be learned (for instance by an opponent system [75]), such that the Pavlovian effect of that negative valence might interfere with the instrumental action (as it did in our behavioral results). The effects of acute tryptophan depletion in the instructed version of the task suggest that serotonin may support some aspects of this opponent signal 17, 22. However, much less is known about the neural mechanisms of aversion and action inhibition than about reward and action invigoration. Recent research suggests that serotonin is indeed involved in circumstances where action inhibition is implemented in response to punishment or its anticipation 17, 22, 37, 75, 76. Action inhibition in the face of reward, as in the stop-signal reaction-time task, has also implicated various regions, including the inferior frontal gyrus [77].

## A role for the prefrontal cortex in overcoming Pavlovian–instrumental conflict

As we have seen, learning the appropriate behavioral response is suboptimal in conditions where Pavlovian and instrumental controllers conflict 18, 21, 23, 24. This poses the question of whether these two systems are segregated in the brain and compete for behavioral control. This notion is supported by evidence that, at a neuronal level, instrumental and Pavlovian responses are supported by different corticostriatal loops [5]. The dorsal striatum is involved in learning and performance of goal-directed and habitual instrumental responding [78]. Sectors of the ventral striatum are more closely affiliated with Pavlovian responding, with the accumbens shell supporting the expression of unconditioned behaviors to rewards and punishments and the accumbens core involved in the expression of appetitive- directed Pavlovian responses 79, 80, 81. The amygdala is also associated with the expression of conditioned responses to punishment 82, 83 and appetitive processing [84]. Indeed, connectivity between the amygdala and the accumbens is implicated in appetitive PIT in animals [85] and humans [28]. However, in our experiments we did not observe any evidence of neural segregation of Pavlovian and instrumental controllers.

Alternatively, or perhaps additionally, Pavlovian and instrumental influences could be more directly intertwined. Consider, for instance, ‘direct’ and ‘indirect’ striatal pathways. These are suggested as promoting, respectively, go choices in light of reward versus no-go choices in light of foregone reward 53, 86, 87. This functional architecture provides a plausible mechanism for instrumental learning of active responses through positive reinforcement (‘go to win’) and passive (avoidance) responses through punishment (‘no go to avoid losing’). However, this architecture cannot account for learning of go choices in the context of punishment (‘go to avoid losing’) or no-go choices in the context of reward (‘no go to win’). Therefore, learning in conditions where Pavlovian and instrumental system conflict requires a supplementary mechanism. As discussed, two-factor theories provide such a supplementary mechanism for the go-to-avoid-losing condition.

For the case of ‘no go to win’, the experiments suggest that prefrontal cortex mechanisms modulated by dopamine are involved in overcoming the Pavlovian bias. Indirect evidence for this comes from the decreased Pavlovian influence during learning after an experimental boost of dopamine levels [18]. Various human experiments have shown an increase or a decrease in (putatively prefrontal) model-based over (striatal) model-free control when dopamine is boosted or depleted, respectively 88, 89. In rats, dopamine achieves these effects by facilitating the operation of prefrontal processes [90], perhaps including components of working memory (on which model-based choice depends [91]) or rule learning 54, 92, 93. This suggests a predominant effect at the level of prefrontal function when the dopaminergic system is systemically manipulated in humans. Whether enhanced dopamine decreases model-free control by improving prefrontal function 54, 90, 92 or by increasing the influence of the prefrontal cortex over subcortical representations 20, 94, 95 needs further study.

Of direct relevance to prefrontal involvement in ‘no go to win’ are increased BOLD responses in the inferior frontal gyrus in trials requiring motor inhibition [21] and theta oscillations in medial frontal areas that are inversely correlated with the influence of the Pavlovian bias on a trial-by-trial basis [23]. The prefrontal cortex may decrease Pavlovian influences through recruitment of the subthalamic nucleus 96, 97, raising a decision threshold within the basal ganglia that prevents execution of a biased decision computed in the striatum 97, 98, 99. Alternatively, the striatum could act to generate a categorical signal that distinguishes activation or inhibition of a given action. After successful learning, this signal shows a clear separation between go and no-go choices (that is enhanced by dopamine); this clarity could depend on the weighting or processing of afferent information from the prefrontal cortex. In either case, via prefrontal prevention of incorrect go choices, participants may experience the richer reward schedule associated with the no-go choice and, over the course of learning, the striatum could eventually represent the appropriate choices, as in the instructed version of the task.

## Concluding remarks

Action and inaction result from interacting instrumental and Pavlovian mechanisms realized at behavioral and neural levels. As a result, action and valence are coupled, so examining the functional complexity of the basal ganglia and their dopaminergic innervation requires them to be manipulated simultaneously. Variants of a task that orthogonalizes action and valence have shown that action dominates valence in the striatum and dopaminergic midbrain. These findings suggest limits to dominant views of dopaminergic and striatal function and invite extensions to include action tendencies (Box 2). A dopaminergic contribution to the control of motivation in instrumental responding is also highlighted, along with its strong effect, putatively at the level of the prefrontal cortex, in regulating the extent to which an obligatory coupling between Pavlovian and instrumental control systems is expressed.

Box 2 **Outstanding questions**

• Which are the neural substrates for the detrimental Pavlovian influence we observed in the learning study (Box 1)?
• Why do so many healthy participants fail to learn in the ‘no go to win’ condition in such a simple task? It is important to entertain and test different computational hypotheses regarding their behavioral inadequacies. It is also worth testing whether the Pavlovian influence observed in this task correlates with putative Pavlovian effects in other tasks measured in the same group of participants.
• Do Pavlovian influences impact model-based and model-free instrumental behavior differently? Richer tasks in which the two forms of instrumental control are more clearly separated are necessary to examine this in detail.
• Is the clear action dependency of the BOLD signal in the striatum evident in some of the many other paradigms that have reported valence during anticipation?
• Can a clear action dependency be translated to electrophysiological or voltammetry measures in animal studies? The bulk of the evidence supporting our views is based on BOLD signal data in humans. Consequently it will be important to develop variations of the complete orthogonalized go/no-go task in animal experiments. The prediction is that neuronal responses of dopaminergic and striatal spiny neurons, as well as dopaminergic transients in the striatum, to instructive reward-predictive cues will be more closely related to the subsequent behavioral response (in terms of activation/inhibition) than to the expected value.
• What is the substrate of aversive prediction and action inhibition? These deserve substantial study because they are much less well understood than appetitive prediction and action invigoration.
• Which are the neural substrates of the observed behavioral effects of systemic dopaminergic manipulations? The dominance of the prefrontal over the expected subcortical effects of dopamine highlights the need for a wider range of methods to manipulate dopaminergic function in humans with higher pharmacological and regional specificity. This should allow the study of tonic and phasic dopamine signaling and the different effects of D1 and D2 receptors in dorsal and ventral striatum.

## Glossary

Action: the behavioral output. Action can be reduced to two categorical extremes: (i) the emission of a behavioral response, or a ‘go’ choice; and (ii) the absence of a particular overt behavioral response, or a ‘no-go’ choice. Sometimes, action and vigor are synonyms, reporting the alacrity of behavioral outputs.
Action-based value: the value associated with the performance of an action at a state.
Conditioned suppression: a form of PIT in which the Pavlovian stimulus predicts the occurrence of a punishment. Presenting it decreases the likelihood and vigor of the instrumental response directed to obtaining reward.
Instrumental control: the determination of behavioral output (the generation or inhibition of overt motor behavior) by an animal or human in the light of the contingency (dependency) between this output and an outcome. A laboratory example is the Skinner box, in which an animal presses a lever to obtain a food pellet.
Omission schedule: an experimental setting in which emitting a particular behavioral response results in the omission of a reward. Negative automaintenance is a type of omission schedule in which a Pavlovian approach to a rewarding stimulus results in omission of a reward.
Pavlovian control: the reflex behavioral output elicited in animals and humans by stimuli that have a contingent relationship with an outcome that can be either a reward or a punishment. The provision or prevention of the outcome is independent of the response. When presented, the outcomes themselves also engender sometimes different Pavlovian responses. The most famous example is the salivation response to the sound of a bell observed in dogs by Pavlov after repeated pairings of the presentation of food with the sound of the bell. The reflex responses differ between species.
Pavlovian–instrumental transfer (PIT): PIT experiments show that Pavlovian responses can influence instrumental control. A typical PIT experiment involves three phases: (i) a Pavlovian training phase, which establishes a stimulus’ ability to predict the delivery of an outcome; (ii) an instrumental training phase in which a particular response is learned to be performed to obtain an outcome; and (iii) a transfer phase in which the (Pavlovian) stimulus is presented while the subject is allowed to perform the instrumental response.
Positive PIT: if the Pavlovian stimulus predicts the occurrence of a reward, presenting it increases the likelihood and vigor of appetitively directed instrumental responses, particularly if the Pavlovian and instrumental outcomes are the same.
Reward-prediction error: the difference between the value obtained when entering a new state or performing an action (reward) and the expected value of being in that state or performing that action. Reward-prediction errors are more complex in tasks that are substantially extended over time.
State-based value: the value associated with being in a given state, averaging over the actions that are taken at that state.
Two-factor theory of active avoidance: this proposes that active avoidance involves two processes or ‘factors’, one Pavlovian and the other instrumental. The Pavlovian factor involves learning that a stimulus predicts an aversive outcome. The instrumental factor involves learning a behavioral response that removes or terminates that stimulus, implying safety.
Valence: the attraction or aversion of an object or situation as a behavioral goal (i.e., whether it is rewarding or punishing). We treat valence and value as synonyms.
